# Incidental findings in CT imaging of coronary artery bypass grafts: results from a Canadian multicenter prospective cohort

**DOI:** 10.1186/s13104-018-3168-1

**Published:** 2018-01-25

**Authors:** I. Boldeanu, J. Perreault Bishop, S. Nepveu, L.-M. Stevens, G. Soulez, T. M. Kieser, A. Lamy, N. Noiseux, C. Chartrand-Lefebvre

**Affiliations:** 1 Department of Radiology, Centre Hospitalier de l’Université de Montréal (CHUM), 1051 Sanguinet Street, Montreal, QC H2X 0C1 Canada; 2 Centre de Recherche du Centre Hospitalier de l’Université de Montréal (CRCHUM), Montreal, Canada; 3 Division of Cardiac Surgery, CHUM, Montreal, Canada; 4 Division of Cardiac Surgery, Libin Cardiovascular Institute of Alberta, Foothills Medical Centre, University of Calgary, Calgary, Canada; 5 Division of Cardiac Surgery, McMaster University and Population Health Research Institute, Hamilton, ON Canada

**Keywords:** Incidental findings, Cardiac computed tomography, Lung, Coronary bypass graft, Lung nodule, Emphysema

## Abstract

**Objective:**

To assess the prevalence and clinical significance of incidental findings identified during computed tomography imaging of coronary artery bypass grafts.

**Results:**

This prospective study includes 144 patients undergoing coronary graft patency assessment using computed tomography. Incidental findings were classified as significant if they were considered to need an immediate action or treatment, short-term work-up or follow-up, or minor. A total of 211 incidental findings were present in 109 (75.7%) patients. Seventy-one incidental findings (33.6%) were cardiac and 140 (66.4%) were extracardiac. Most common cardiac incidental findings were atrial dilatation [39 patients, 48 incidental findings (67.6%)] and aortic valve calcifications (7 patients, 9.9%). Among the 140 extracardiac incidental findings, the most common were lung nodules (51 patients, 54 nodules, 38.6%), and emphysema (21 patients, 15%). Thirty-six (25.7%) extracardiac incidental findings were significant and notably, 23 (63.9%) were lung nodules. Follow-up was recommended in 37 cases, among which all patients with significant lung nodules (23 patients, 62.2%). In conclusion, most common computed tomography incidental findings in patients with coronary grafts were lung nodules and emphysema.

**Electronic supplementary material:**

The online version of this article (10.1186/s13104-018-3168-1) contains supplementary material, which is available to authorized users.

## Introduction

Heart disease remains the main cause of hospitalization and death in industrialized countries, with a significant health care burden [1]. In specific cases, coronary artery bypass graft surgery (CABG) is the therapeutic approach of choice in order to restore adequate blood flow to an ischemic heart and prevent further cardiovascular complications [2]. It is estimated 300,000 CABG surgeries are performed per year in the United States alone [3]. CABG can be performed either using cardiopulmonary bypass (on-pump) [4–6] or off-pump techniques [2, 7].

In contrast to other cardiac imaging modalities such as echocardiography, nuclear medicine studies or even cardiac magnetic resonance, cardiac computed tomography (CT) allows to visualize the surrounding lungs, mediastinum, pleura and upper abdominal cavity in addition to the heart. The assessability of extracardiac anatomy and incidental findings (IF) gives the opportunity for alternative diagnoses to the patient’s symptoms, or for the detection of lesions otherwise silent at the time of the examination [8, 9]. IF may include findings from benign and nonsignificant incidentals such as benign chest granulomatous disease to malignant pulmonary nodules. The overall prevalence of extracardiac findings is high. A meta-analysis reported a pooled prevalence of 44% in patients undergoing cardiac CT examinations, most of which were performed for coronary calcium scoring or coronary CT angiography [9].

However, prevalence of IF may vary according to the specific indication or population in which cardiac CT is used. Moreover, CT scanning for coronary calcium scoring or coronary CT angiography is limited to the heart and involves limited portions of the extracardiac anatomy. In contrast, CT acquisition performed for CABG evaluation involves more lung volume to be assessable, since scanning will cover from the length of the mammary arteries as well as the heart. In addition, some individuals undergoing calcium scoring or coronary CT angiography are only at low risk for lung cancer. Most patients with CABG, on the other hand, are often current or former smokers at increased risk for IF, especially pulmonary nodules.

This study includes 144 consecutive CABG patients undergoing graft patency prospective assessment using CT angiography at 1-year postoperative follow-up. It aims to assess the prevalence of both benign and significant cardiac and extra-cardiac IF in the specific population of patients with CABG.

## Main text

### Methods

#### Study population

The CABG Off or On Pump Revascularization Study (CORONARY) is a prospective randomized trial (N = 4752 patients) comparing CABG surgery performed on- or off-pump (NCT00463294, registration April 18, 2007) [10]. A secondary study of CORONARY was held in three Canadian centers (N = 157 patients) with the objective to evaluate CABG patency after 1-year follow-up using CT [7]. The present study includes the initial 144 patients [122 males, 22 females, median age 69.0 (64.0–75.0) years] recruited in this secondary study.

#### CT imaging

##### CT acquisition

In the three institutions, a total of  three scanners were used: a 256-slice CT scanner (Brilliance iCT, Philips Healthcare, Cleveland, OH, USA) in 117 patients (site 1), and two 64-slice scanners (Aquilion64, Toshiba Medical Systems, Tochigi, Japan; and GE LightSpeed VCT; GE Healthcare, USA), in 16 (site 2) and 11 (site 3) patients, respectively. Image acquisition used a matrix of 512 × 512 and a field-of-view (FOV) of 250 mm. Scan voltage was 120 kV, and gantry speed varied from 270 to 400 ms. Slice thickness was 0.5–0.625 mm. Prospective ECG-gating was used when available, for heart rate < 70 bpm; if not, retrospective ECG-gating was used. Scans were performed during shallow inspiration breath-hold, with scan range from the clavicles to the lung bases just caudal to the heart in order to visualize the heart as well as the internal mammary arteries. The contrast agent was administered at 5 ml/s.

##### Image reconstruction and postprocessing

Image reconstruction used both standard (smooth) and lung kernels. In sites 2 and 3, a larger reconstructed FOV of 320–500 mm was also used, with lung kernel. At the time of the study, filtered back projection was used for image reconstruction. One observer (CCL, 15-year experience in cardiovascular CT) reviewed all images and performed CABG patency image analysis in a central core lab (site 1).

#### IF classification

Incidental findings were recorded prospectively. IF were defined as unexpected observations of potential clinical significance and unrelated to the main purpose of the CORONARY CT angiography secondary study (i.e. graft patency assessment). IF were classified as significant if they were considered to need a clinical action, or minor. If deemed significant, the findings were further described regarding the type of action needed (treatment, work-up or follow-up) and the time period in which these actions were to be performed. The Fleischner Society pulmonary nodule recommendations were used to evaluate and manage solid and subsolid incidental pulmonary lung nodules [11, 12]. The time periods were categorized as 0–0.9 month (requiring immediate action), 1–2.9, 3–5.9, 6–9.9 or 9–12 months. Imaging follow-up modalities included CT, magnetic resonance, ultrasonography, or other. The findings were also categorized in respect to their anatomical location, as well as the type of abnormality. Native distal bed coronary artery stenoses were collected in the CORONARY CT angiography study, but however were not considered as incidental in this population of patients with coronary disease.

#### Statistical analysis

Data were expressed as median and interquartile range (Q1–Q3) for continuous variables, and as frequencies (percentages) for categorical variables. The Mann–Whitney U test was used for continuous variables. For categorical variables, the Fisher exact test was used. A two-tailed p value < 0.05 was considered significant. Statistical analyses were performed using SPSS (version 22, Inc, Chicago, Illinois).

### Results

All CT scans were completed successfully. No scan was excluded from the analysis. Median z-axis scan coverage was 250.0 (235.25–272.0) mm. See Table 1 for additional scan-related parameters.

**Table 1:** Patient characteristics and scan-related parameters

Patient characteristics (n = 144 patients)	
Men/women	122/22
Age (years) [median (Q1–Q3)]	69.0 (64.0–75.0)
Presence of IF (n, %)	109 (75.7%)
BMI (kg/m^2^) [median (Q1–Q3)]	27.40 (25.48–30.42)
Smoker^a^ (n, %)	105 (72.9%)
Hypertension (n, %)	126 (87.5%)
Diabetes (n, %)	47 (32.6%)
Renal insufficiency (n, %)	11 (7.6%)
CHF (n, %)	5 (3.5%)
EuroScore	
1 (n, %)	17 (11.8%)
2 (n, %)	27 (18.8%)
3 (n, %)	45 (31.3%)
4 (n, %)	31 (21.5%)
5 (n, %)	19 (13.2%)
6 (n, %)	4 (2.8%)
7 (n, %)	0 (0.0%)
8 (n, %)	1 (0.7%)
Scan-related parameters	
Mean heart rate during scan (bpm) [median (Q1–Q3)]	58.0 (53.0–64.0)
Prescan betablocker administration (n, %)	65 (45.1%)
Prescan nitroglycerin administration (n, %)	137 (95.1%)
Contrast agent (ml) [median (Q1–Q3)]	102.0 (100.0–105.75)
Scan coverage (mm) [median (Q1–Q3)]	250.0 (235.25–272.0)
DLP (mGy-cm) [median (Q1–Q3)]	683.0 (469.0–897.0)
Effective dose^b^ (mSv) [median (Q1–Q3)]	9.6 (6.6–12.6)
ECG gating (prospective/retrospective)	134/10

*BMI* Body mass index, *bpm* beats per minute, *CHF* congestive heart failure, *EuroSCORE* European System for Cardiac Operative Risk Evaluation, *IF* incidental findings, *DLP* dose-length product for CT angiography

^a^Current and former smokers were considered as smokers

^b^The effective radiation dose was estimated by the product of the DLP and a conversion coefficient for the thorax (k = 0.014 mSv mGy^−1^ cm^−1^)

#### Global incidental findings

Among the 144 patients, a total of 211 IF were present in 109 (75.7%) patients. Thirty-five patients (24.3%) presented no IF. Among the 211 IF recorded, 71 (33.6%) were cardiac IF in 52 patients and 140 (66.4%) were extracardiac IF in 87 patients (some patients presented both) (Table 2).

**Table 2:** Prevalence of incidental cardiac and extracardiac findings

	Total IF	% of cardiac or extracardiac IF	% of all IF	Significant IF
Incidental cardiac findings				
Left atrial dilatation	34	47.9	16.1	
Right atrial dilatation	14	19.7	6.6	
Aortic valve calcifications	7	9.9	3.3	
Lipomatous metaplasia (chronic myocardial infarction)	3	4.2	1.4	
Pericardial effusion	3	4.2	1.4	
Left ventricular hypertrophy	2	2.3	1.0	1
Atrial septal aneurysm	1	1.4	0.5	
Left atrial thrombus	1	1.4	0.5	
Other	6	8.5	2.8	
Total	71	100.0	33.6	1
Incidental extracardiac findings				
Pulmonary nodules	54	38.6	25.6	23
Emphysema	21	15.0	10.0	
Pleural effusion/thickening	6	4.2	2.8	
Thoracic aorta dilatation	5	3.6	2.4	
Enlarged mediastinal lymph nodes	4	2.9	1.9	3
Subclavian artery stenosis	4	2,9	1.9	
Main pulmonary artery dilatation	2	1.4	1.0	
Brachiocephalic artery stenosis	1	0.7	0.5	
Pulmonary infiltrates	1	0.7	0.5	1
Hiatal hernia	17	12.1	8.1	
Hepatic lesions	10	7.1	4.7	6
Abdominal aorta aneurysm	1	0.7	0.5	
Adrenal gland adenoma	1	0.7	0.5	
Thyroid nodules	7	5.0	3.3	3
Breast abnormalities	2	1.4	1.0	
Other	4	2.9	1.9	
Total	140	100	66.4	36

Among cardiac findings, the most common findings were left or right atrial dilatation (or both) [39 patients, total of 48 IF (67.6% of cardiac IF)] and aortic valve calcifications (7 patients, 9.9% of cardiac IF) (Table 2). Other cardiac findings included left ventricular hypertrophy, atrial septal aneurysm, pericardial effusion, left atrial thrombus and intra-atrial shunt. Additional file 1 describes the case of the intra-atrial shunt. A case of lipomatous metaplasia of the left ventricular wall suggestive of a chronic myocardial infarction is shown in Additional file 2. A cardiac ultrasound was recommended for the case of left ventricular hypertrophy. Other findings were already known by the treating team or were the subject of contemporary work-up.

A total of 140 extracardiac IF were found in 87 (60.4%) of our 144 patients. The most common were pulmonary nodules [51 patients; 54 nodules (38.6% of extracardiac IF)], followed by emphysema (21 patients, 15.0%). Thirty-six (25.7%) extracardiac IF were categorized as significant. Notably, near two-thirds (23, 63.9%) of significant extracardiac IF were pulmonary nodules. Median size was 5.65 (4.00–7.75) mm; range was 3–11 mm). CT follow up was recommended for the 23 significant pulmonary nodules. Extrathoracic IF involved the abdominal (29 cases, 20.7% of all extracardiac IF), cervical (7 cases, 5.0%) and mammary (2 cases, 1.4%) regions (Table 2). Recommendations for clinically significant extracardiac findings, other than pulmonary nodules, were abdominal, cervical or breast ultrasonography.

#### Significant incidental findings

Thirty-seven significant incidental findings occurred in 36 (25%) patients. Pulmonary nodules (23 patients, 62.2% of significant IF) and hepatic lesions (6 patients, 16.2%) were the most common. Additional files 3 and 4 describe cases of incidental lung and hepatic findings. Cases of thyroid nodule (3 patients, 8.1%) and enlarged mediastinal lymph nodes (3 patients, 8.1%) were also observed, as well as a single case of left ventricular hypertrophy (2.7%) and one of pulmonary infiltrate (2.7%). Most follow-up recommendations involved CT (73%). Ultrasonography was suggested for all other cases (23%). Time periods for follow-up are described in Table 3.

**Table 3:** Prevalence of incidental findings requiring action according to type of work-up and time period suggested for follow-up (n = 37 cases of IF)

Type of IF (n, %)	
Pulmonary nodule	23 (62.2)
Pulmonary infiltrate	1 (2.7)
Enlarged mediastinal lymph nodes	3 (8.1)
Left ventricular hypertrophy	1 (2.7)
Thyroid nodule	3 (8.1)
Hepatic lesion	6 (16.2)
Type of recommended modality for follow-up (n, %)	
Computed tomography	27 (73.0)
Ultrasonography	10 (27.0)
Time frame of recommended follow-up (n, %), months	
0–0.9	3 (8.1)
0–2.9	11 (29.7)
3–5.9	14 (37.8)
6–8.9	3 (8.1)
9–11.9	6 (16.2)

#### Comparison between patients with and without IF

Patients of both groups had comparable baseline demographics and selected clinical variables, except for a significantly lower prevalence of congestive heart failure in patients who presented incidental findings (p = 0.041). A description of the patient characteristics by incidental finding status is provided in Additional file 5. Among patients with lung nodules versus without, proportion of current or former smokers were 60.9% (25/41) and 77.6% (80/103), respectively (p = 0.060). Among patients with significant lung nodules versus without any nodules, the proportions of current or former smokers were 70.0% (16/23) and 76.8% (73/95), respectively (p = 0.590).

### Discussion

This is a multicenter study involving 144 CABG patients, with prospective assessment of cardiac CT scans for minor or significant IF. A total of 211 IF were found in 109 (75.7%) patients. Most IF (66.4%) were extracardiac IF. Pulmonary nodules accounted for more than half of the significant IF. Hepatic lesions were the second most frequent significant IF.

In a recent meta-analysis, the pooled prevalence of cardiac CT patients with at least one extracardiac IF was 44% [9]. Prevalence in the primary studies of this meta-analysis showed a high variability, ranging from 14.8% [13] to 79.4% [9, 14]. This variability is probably due to the different patient populations studied. Of note, extracardiac IF in our population of CABG patients had a prevalence of 60.4%. Study design, CT protocol, patient characteristics and level of attention to incidental findings may also explain this variability [9, 15]. Another cause to this heterogeneity is the interobserver discordance as to the presence or significance of IF [16].

Pulmonary nodules were the most frequent significant IF in our study, accounting for 62.2% of all significant IF, and occurring in 23 (16.0%) of patients. Previous studies reported that prevalence of significant pulmonary nodules in cardiac CT were 0.9% [13] to 16.5% [17]. Excluding electron-beam CT from its dataset, the calculated weighted prevalence of significant pulmonary nodules in Earls’ review paper is 3.8% [15]. Another meta-analysis reported pooled prevalences of 16% for significant IF and of 0.7% for incidental cancer, of which most were lung cancer [9]. In view of these rates of significant IF and incidental cancers, as well as of results of lung cancer screening trials with CT showing reduction of mortality due to lung cancer [18, 19], many authors advocate that all images from cardiac CT should be reviewed by an expert thoracic CT imager.

Only two former studies assessed IF in the specific population of CABG patients. A retrospective study [20] of 259 patients assessed with 16-slice CT during the immediate postoperative period, of which 40 patients were also assessed prospectively after a mean follow-up of 12.7 months. 13.1% of their patients had a significant noncardiac IF. Especially, prevalence of significant pulmonary nodules was 3.5%. Another retrospective study [21] included 223 patients with CABG, assessed with 64-slice CT outside the postoperative period. The prevalence of pulmonary nodules was 10.8%. In comparison, our study had a prospective design, with prevalence of significant pulmonary nodules of 16%. Description of studies assessing incidental findings in cardiac CT is available in Additional file 6.

In conclusion, our multicenter prospective study involving 144 CABG patients assessed with CT shows a 25% prevalence of significant extracardiac IF, most of which were pulmonary nodules. With these results in mind, as well as in view of the data from recent lung cancer screening trials and the precautionary principle, cardiac CT interpretation in CABG patients should include the assessment of all images by an expert thoracic CT imager.

## Limitations

Our study involves limited follow-up data. Second, our analysis does not include health care cost evaluation. Finally, since large FOV were used only in sites 2 and 3, prevalence rates could be underestimated for peripheral lung nodules. Strengths of the study involves its multicenter prospective design, as well as the specific prospective definition of the population studied.

## Additional files

**Additional file 1.** Intra-atrial shunt. A 60-year-old man presenting with a right-to-left intra-atrial shunt (black arrow), as shown by a jet flow of non-enhanced blood in the left atrium (arrow). A 7-mm ostium secundum atrial septal defect with bidirectional flow was also confirmed at trans-esophageal echocardiography. The patient underwent minimally invasive transcatheter closure with a septal occluder.

**Additional file 2.** Lipomatous metaplasia. A 62-year-old man presenting with lipomatous metaplasia in the lateral wall of the left ventricle, suggestive of myocardial infarction (arrows).

**Additional file 3.** Pulmonary nodule. 256-slice computed tomography angiography with prospective ECG-gating; 3D volume rendering (A) and axial slice at the level of the left lung apex (lung reconstruction kernel, 5 mm thickness (B). A, In situ left internal mammary artery with saphenous bridge graft (SVB) to the left anterior descending artery and to a diagonal branch, in a 73-year-old man, with 1-year postoperative follow-up. The LIMA (white arrow) is seen from its origin from the left subclavian artery. The SVB distributes the flow to both the LAD and the diagonal branch. There is also an aortocoronary saphenous vein graft to a distal obtuse marginal branch (black arrow). B, Incidental nodule (10 mm) in the apex of the left lung (black arrow). Follow-up scans were recommended. The nodule was stable at 12-month follow-up.

**Additional file 4.** Hepatic lesion. 256-slice computed tomography angiography with prospective ECG-gating; 3D volume rendering (A) and axial slice at the level of the left lobe of the liver (0.8 slice thickness (B). A, In situ left internal mammary artery to the left anterior descending artery, in a 81-year-old man, with 1-year postoperative follow-up. The LIMA (A, white arrow) is seen from its origin from the left subclavian artery. B, Incidental lesion is seen in the 2nd segment of the left lobe of the liver (18 mm) (black arrow). Lesion attenuation was 20-25 Hounsfield units, and could be a solid lesion. An ultrasound was recommended.

**Additional file 5.** Summary of patient characteristics by incidental finding status.

**Additional file 6.** Comparison of incidental findings studies.

## Abbreviations

CABG: coronary artery bypass graft
CT: computed tomography
IF: incidental findings
FOV: field-of-view
