# Supplementary material for: Incidental findings in CT imaging of coronary artery bypass grafts: results from a Canadian multicenter prospective cohort
Source: BMC Res Notes. 2018 Jan 25;11:72. doi: 10.1186/s13104-018-3168-1 (PMC5784672; doi:10.1186/s13104-018-3168-1)
Supplement: Supplementary file 1 — Additional file 1. Intra-atrial shunt. A 60-year-old man presenting with a right-to-left intra-atrial shunt (black arrow), as shown by a jet flow of non-enhanced blood in the left atrium (arrow). A 7-mm ostium secundum atrial septal defect with bidirectional flow was also confirmed at trans-esophageal echocardiography. The patient underwent minimally invasive transcatheter closure with a septal occluder. [file 13104_2018_3168_MOESM1_ESM.pptx]

## Slide 1
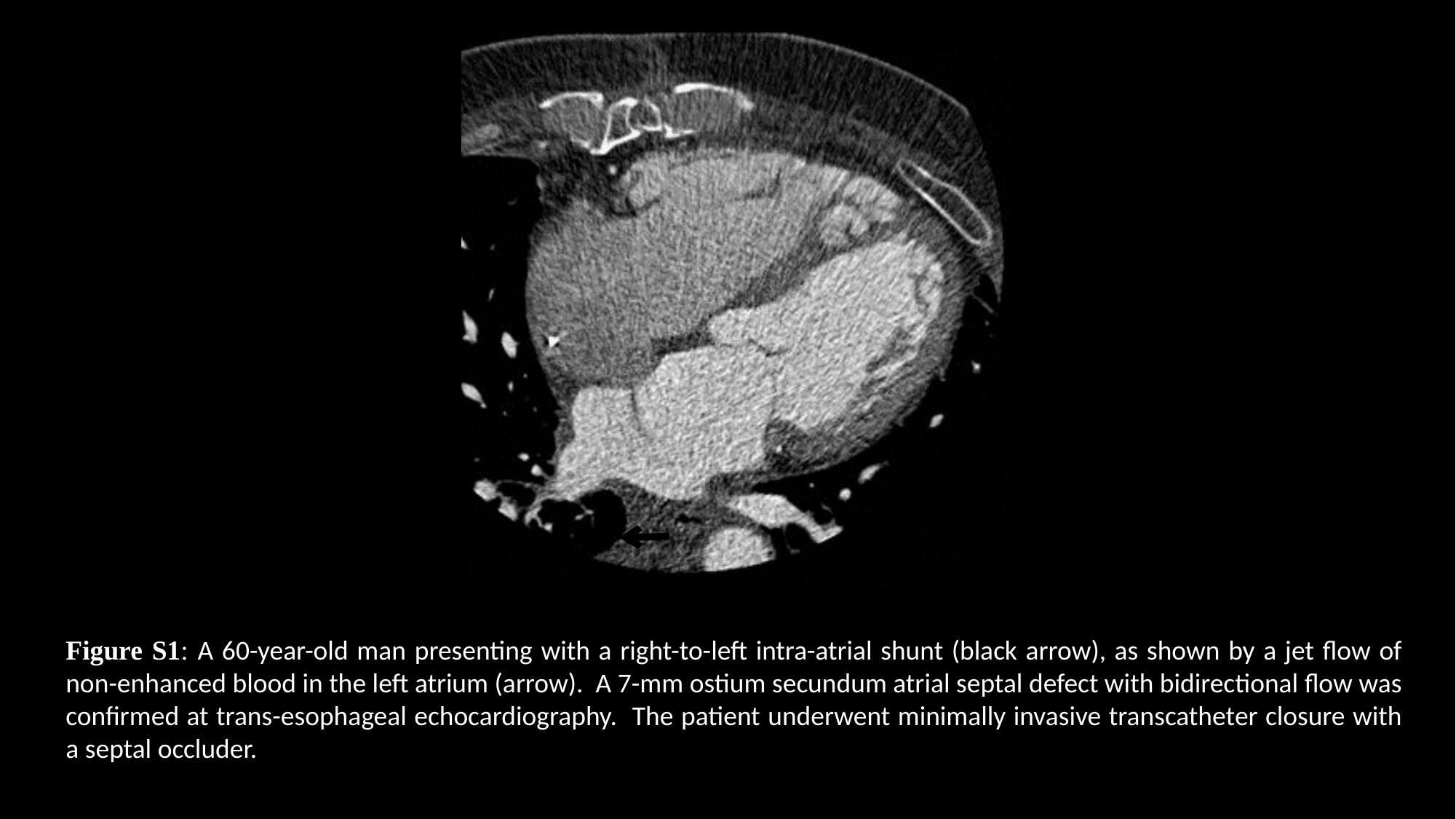

B
Figure S1: A 60-year-old man presenting with a right-to-left intra-atrial shunt (black arrow), as shown by a jet flow of non-enhanced blood in the left atrium (arrow). A 7-mm ostium secundum atrial septal defect with bidirectional flow was confirmed at trans-esophageal echocardiography. The patient underwent minimally invasive transcatheter closure with a septal occluder.
