# Supplementary material for: Incidental findings in CT imaging of coronary artery bypass grafts: results from a Canadian multicenter prospective cohort
Source: BMC Res Notes. 2018 Jan 25;11:72. doi: 10.1186/s13104-018-3168-1 (PMC5784672; doi:10.1186/s13104-018-3168-1)
Supplement: Supplementary file 2 — Additional file 2. Lipomatous metaplasia. A 62-year-old man presenting with lipomatous metaplasia in the lateral wall of the left ventricle, suggestive of myocardial infarction (arrows). [file 13104_2018_3168_MOESM2_ESM.pptx]

## Slide 1
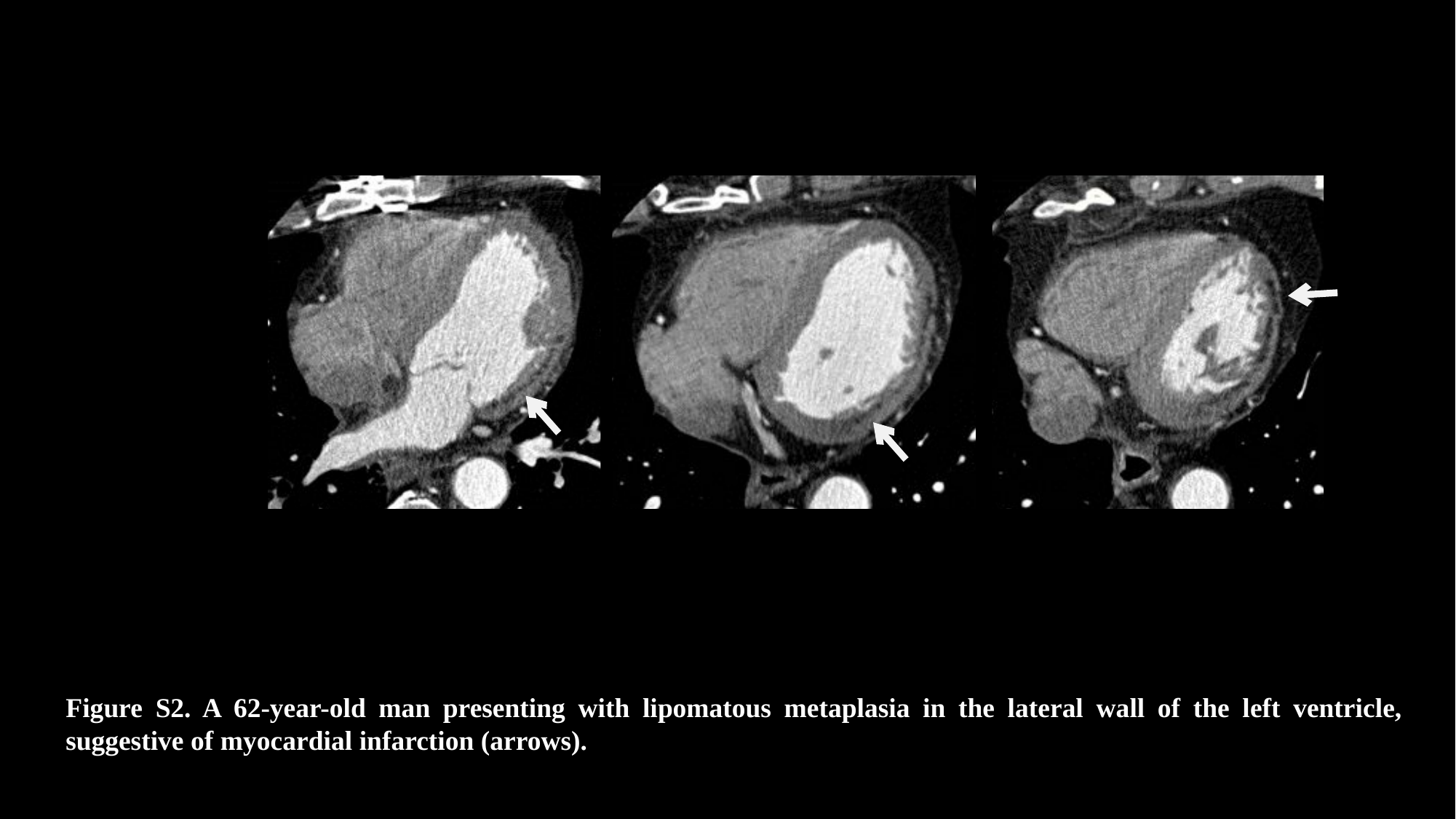

B
Figure S2. A 62-year-old man presenting with lipomatous metaplasia in the lateral wall of the left ventricle, suggestive of myocardial infarction (arrows).
