# Supplementary material for: Incidental findings in CT imaging of coronary artery bypass grafts: results from a Canadian multicenter prospective cohort
Source: BMC Res Notes. 2018 Jan 25;11:72. doi: 10.1186/s13104-018-3168-1 (PMC5784672; doi:10.1186/s13104-018-3168-1)
Supplement: Supplementary file 3 — Additional file 3. Pulmonary nodule. 256-slice computed tomography angiography with prospective ECG-gating; 3D volume rendering (A) and axial slice at the level of the left lung apex (lung reconstruction kernel, 5 mm thickness (B). A, In situ left internal mammary artery with saphenous bridge graft (SVB) to the left anterior descending artery and to a diagonal branch, in a 73-year-old man, with 1-year postoperative follow-up. The LIMA (white arrow) is seen from its origin from the left subclavian artery. The SVB distributes the flow to both the LAD and the diagonal branch. There is also an aortocoronary saphenous vein graft to a distal obtuse marginal branch (black arrow). B, Incidental nodule (10 mm) in the apex of the left lung (black arrow). Follow-up scans were recommended. The nodule was stable at 12-month follow-up. [file 13104_2018_3168_MOESM3_ESM.pptx]

## Slide 1
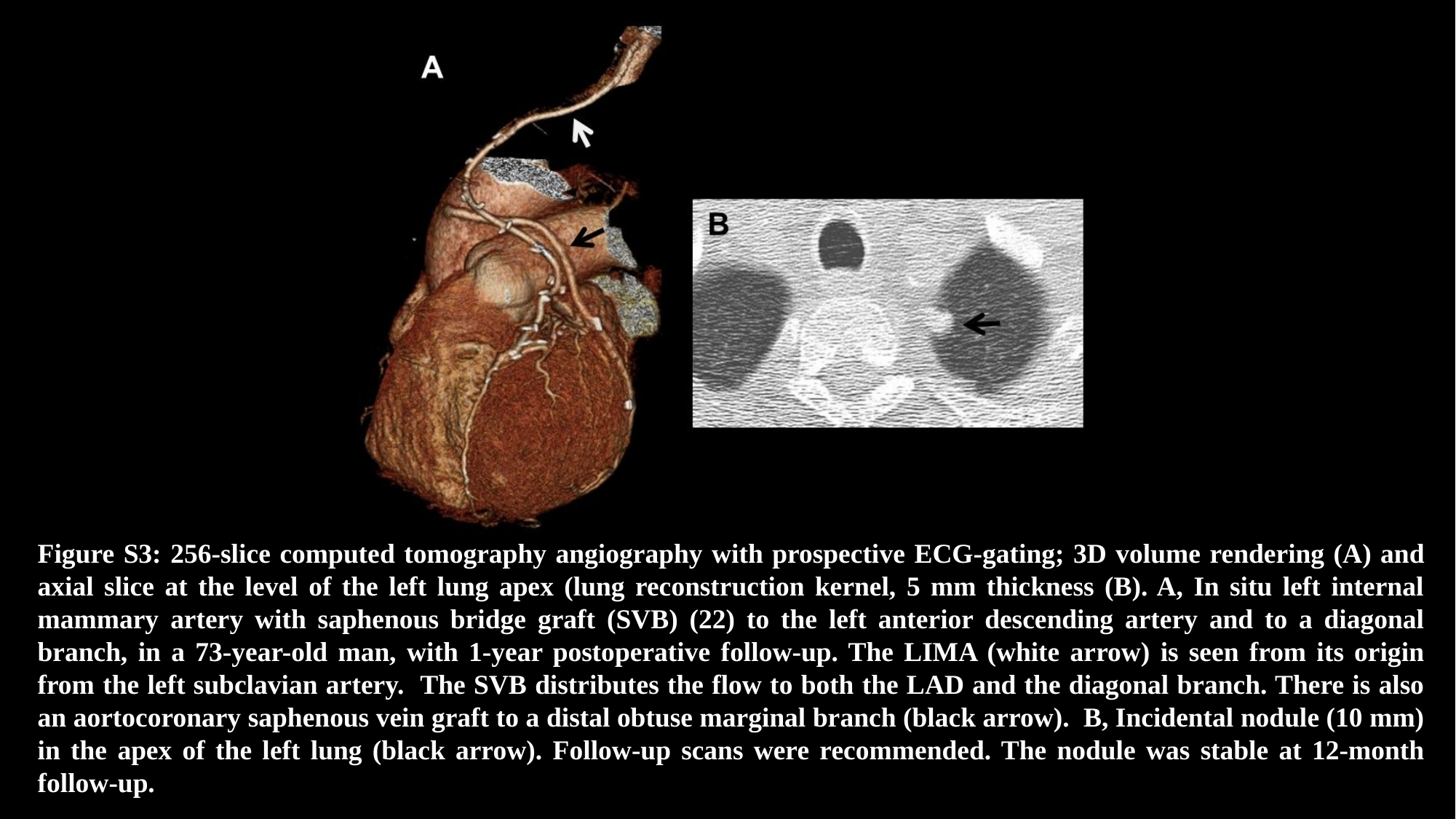

Figure S3: 256-slice computed tomography angiography with prospective ECG-gating; 3D volume rendering (A) and axial slice at the level of the left lung apex (lung reconstruction kernel, 5 mm thickness (B). A, In situ left internal mammary artery with saphenous bridge graft (SVB) (22) to the left anterior descending artery and to a diagonal branch, in a 73-year-old man, with 1-year postoperative follow-up. The LIMA (white arrow) is seen from its origin from the left subclavian artery. The SVB distributes the flow to both the LAD and the diagonal branch. There is also an aortocoronary saphenous vein graft to a distal obtuse marginal branch (black arrow). B, Incidental nodule (10 mm) in the apex of the left lung (black arrow). Follow-up scans were recommended. The nodule was stable at 12-month follow-up.
