# Supplementary material for: Incidental findings in CT imaging of coronary artery bypass grafts: results from a Canadian multicenter prospective cohort
Source: BMC Res Notes. 2018 Jan 25;11:72. doi: 10.1186/s13104-018-3168-1 (PMC5784672; doi:10.1186/s13104-018-3168-1)
Supplement: Supplementary file 4 — Additional file 4. Hepatic lesion. 256-slice computed tomography angiography with prospective ECG-gating; 3D volume rendering (A) and axial slice at the level of the left lobe of the liver (0.8 slice thickness (B). A, In situ left internal mammary artery to the left anterior descending artery, in a 81-year-old man, with 1-year postoperative follow-up. The LIMA (A, white arrow) is seen from its origin from the left subclavian artery. B, Incidental lesion is seen in the 2nd segment of the left lobe of the liver (18 mm) (black arrow). Lesion attenuation was 20-25 Hounsfield units, and could be a solid lesion. An ultrasound was recommended. [file 13104_2018_3168_MOESM4_ESM.pptx]

## Slide 1
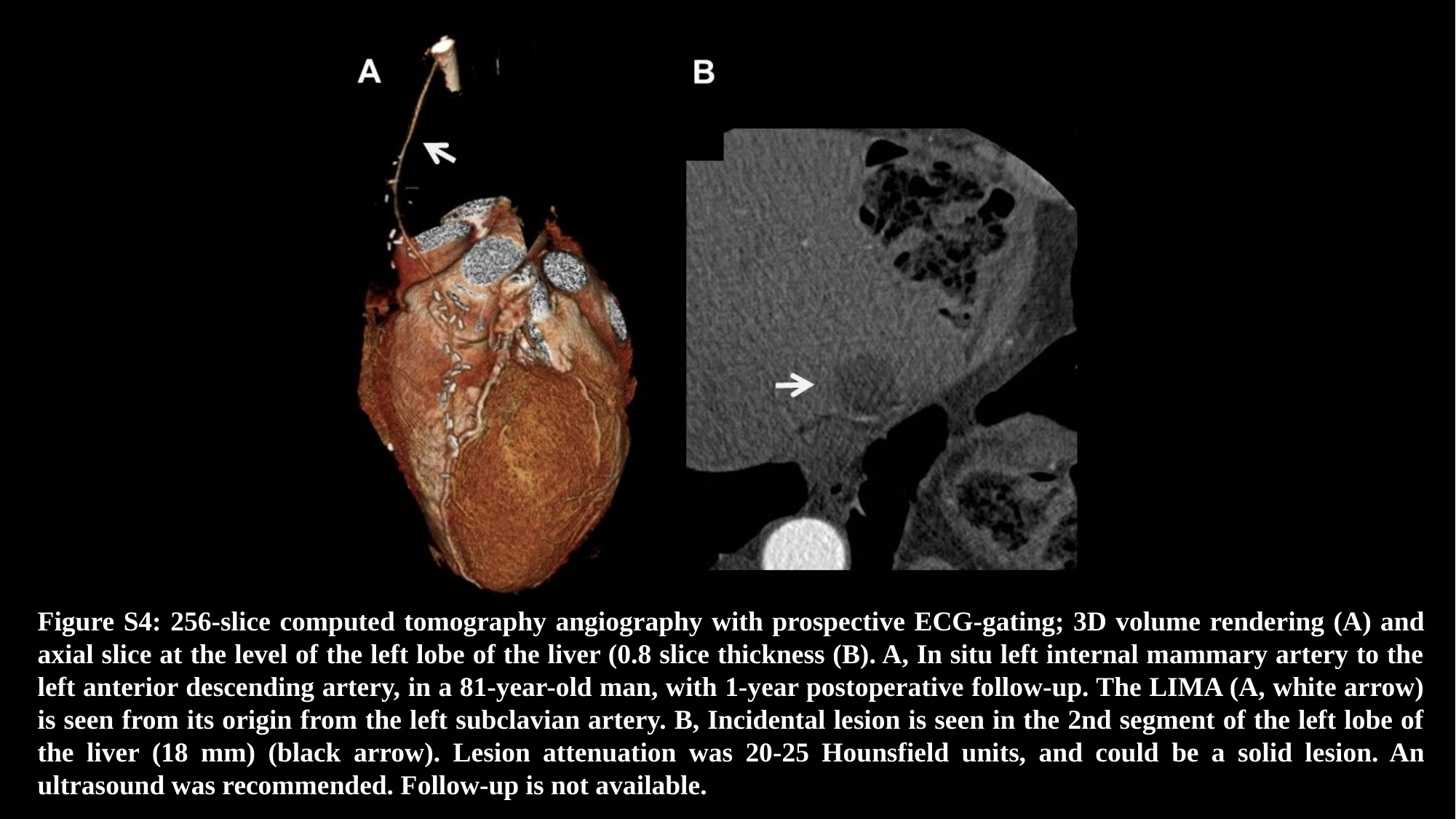

B
Figure S4: 256-slice computed tomography angiography with prospective ECG-gating; 3D volume rendering (A) and axial slice at the level of the left lobe of the liver (0.8 slice thickness (B). A, In situ left internal mammary artery to the left anterior descending artery, in a 81-year-old man, with 1-year postoperative follow-up. The LIMA (A, white arrow) is seen from its origin from the left subclavian artery. B, Incidental lesion is seen in the 2nd segment of the left lobe of the liver (18 mm) (black arrow). Lesion attenuation was 20-25 Hounsfield units, and could be a solid lesion. An ultrasound was recommended. Follow-up is not available.
