# Supplementary material for: Incidental findings in CT imaging of coronary artery bypass grafts: results from a Canadian multicenter prospective cohort
Source: BMC Res Notes. 2018 Jan 25;11:72. doi: 10.1186/s13104-018-3168-1 (PMC5784672; doi:10.1186/s13104-018-3168-1)
Supplement: Supplementary file 5 — Additional file 5. Summary of patient characteristics by incidental finding status. [file 13104_2018_3168_MOESM5_ESM.docx]

| **Additional file 5.** Summary of patient characteristics by incidental finding status | | | |
| --- | --- | --- | --- |
|  | | | |
| **Variable** | **No Incidental Findings (n=26)** | **Presence of Incidental Findings (n=118)** | **P value^a^** |
| Age, mean (SD), y | 69.6 (± 6.4) | 69.7 (± 6.4) | 0.60 |
| Men, % | 85.7 | 84.4 | 0.851 |
| BMI > 25, % | 88.2 | 77.1 | 0.157 |
| Renal insufficiency, % | 0.0 | 9.3 | 0.215 |
| Congestive heart failure, % | 11.5 | 1.7 | 0.041 |
| Diabetes, % | 34.6 | 32.2 | 0.812 |
| Hypertension, % | 88.5 | 87.3 | >.99 |
| Current and former Smoker, % | 77.1 | 71.6 | 0.518 |
| Abbreviations: BMI, body mass index.  ^a^Mann-Whitney U Test for means; *X^2^* for proportions. | | | |
