# Supplementary material for: Incidental findings in CT imaging of coronary artery bypass grafts: results from a Canadian multicenter prospective cohort
Source: BMC Res Notes. 2018 Jan 25;11:72. doi: 10.1186/s13104-018-3168-1 (PMC5784672; doi:10.1186/s13104-018-3168-1)
Supplement: Supplementary file 6 — Additional file 6. Comparison of incidental findings studies. [file 13104_2018_3168_MOESM6_ESM.doc]

| **Additional file 6.** Comparison of incidental findings studies | | | | | | |  |  |
| --- | --- | --- | --- | --- | --- | --- | --- | --- |
|  |  |  |  |  |  |  |  |  |
| **Incidental Findings Study** | **Baseline characteristics^a^** | **Number of patients** | **Overall Incidental Findings Prevalence, %^b^** | **Prevalence of significant Incidental Findings; Recommended follow up examination, %** | **Prevalence of pulmonary nodules, % (with and without follow up recommended)** | **Prevalence of extracardiac incidental findings, %.** | **Target CT assessment** | **Number of patients with CABG** |
| Burt et al., 2008; | Mean age, 66 y; Men, 48%; Smokers, 67% | 459 | 41 | 23 | 19 / 26 | 36 | Cardiac CT (calcium scoring), in asymptomatic subjects without CAD | None |
| Foley, Hamaad, El-Gendi, & Leyva, 2010; | Mean age, 64 y; Men, -, - | 100 | NA | NA | NA / 14 | 39 | Patients suspected for PE, undergoing CT pulmonary angiography | NA |
| Koonce, Schoepf, Nguyen, Northam, & Ravenel, 2009^c^; | Mean age, 58 y; Men, 61%, - | 1764 | 25 | 18 | 11 / 11 | 25 | Investigate ECF using CT based on indication and impact on patient management (CS, CTA, PVS, CABG) | 223 (13%) |
| Lee et al., 2010^c^; | Mean age, 54; Men, 70%, Smokers, 7% | 151 | 43 | 31 | 9 / 26 | 43 | Retrospectively study ECF in patients undergoing coroCT during a 7-year period | NA |
| Mueller, J., Jeudy, J., Poston, R., & White, C. S. (2007). | NA | 259 | 20 | 20 | 2 / 4 | 13 | Retrospective assessment of prevalence of IF after CABG | 259 (100%) |
| Gufler, H., Schulze, C. G., & Wagner, S. (2014) | Mean age, 82; Men, 53%; Smokers, 34%. | 131 | 100 | 24 | 5 / 63 | 22 | Patients referred for CTA to assess suitability for TAVR | NA |
| *Our study* | Mean age, 70; Men, 85%, Smokers, 73%. | 144 | 82 | 25 | 16 / 19 | 60 | CABG patency assessment following on- or off-pump CPB | 144 (100%) |
| ^a^ Smokers encompass both current and former smokers; ^b^ All prevalence statistics are calculated referring to number of patients; ^c^ Only extracardiac findings were reported; ^d^ Percentages regarding number of IF, not number of patients; CAD, coronary artery disease; CPB; cardiopulmonary bypass; ECF, extracardiac findings; PE, pulmonary embolism; TAVR, transcatheter aortic valve replacement; NA, not available. | | | | | | |  |  |
